# Effects of a telework environment intervention on work setup behaviors and somatic symptoms among Japanese teleworkers: a secondary analysis of the TELEWORK cluster randomized controlled trial

**DOI:** 10.1093/joccuh/uiag042

**Published:** 2026-07-26

**Authors:** Satoru Kanamori, Aya Wada, Jihoon Kim, Wataru Umishio, Takahiko Yoshimoto, Rumi Tsukinoki, Natsumi Yoshioka, Kaori Yoshiba, Masahiko Gosho, Yoshio Nakata, Yuko Kai

**Affiliations:** Graduate School of Public Health, Teikyo University, 2-11-1 Kaga, Itabashi-ku, Tokyo, 173-8605, Japan; Department of Preventive Medicine and Public Health, Tokyo Medical University, Tokyo, Japan; Physical Fitness Research Institute, Meiji Yasuda Life Foundation of Health and Welfare, Tokyo, Japan; Institute of Health and Sport Sciences, University of Tsukuba, Ibaraki, Japan; Department of Architecture and Building Engineering, School of Environment and Society, Institute of Science Tokyo, Tokyo, Japan; Department of Hygiene, Public Health and Preventive Medicine, Showa Medical University School of Medicine, Tokyo, Japan; Department of Public Health Nursing, Institute of Science Tokyo, Tokyo, Japan; Physical Fitness Research Institute, Meiji Yasuda Life Foundation of Health and Welfare, Tokyo, Japan; Physical Fitness Research Institute, Meiji Yasuda Life Foundation of Health and Welfare, Tokyo, Japan; Department of Biostatistics, Institute of Medicine, University of Tsukuba, Tsukuba, Ibaraki, Japan; Institute of Health and Sport Sciences, University of Tsukuba, Ibaraki, Japan; Physical Fitness Research Institute, Meiji Yasuda Life Foundation of Health and Welfare, Tokyo, Japan

**Keywords:** teleworking, ergonomics, behavioral sciences, somatic symptoms, randomized controlled trials as topic, occupational health

## Abstract

**Objectives:**

Home workspace ergonomics may influence telework-related somatic symptoms; however, evidence regarding interventions promoting teleworkers’ self-directed environment setup remains limited. We examined the effects of a behavioral science-based educational intervention on telework environment setup behaviors and somatic symptoms among Japanese employees.

**Methods:**

This was a secondary analysis of the TELEWORK cluster randomized controlled trial (UMIN000053861). Workplace departments were randomized as clusters (1:1) to intervention/control. The 12-week program comprised educational emails, short videos, individualized feedback sheets, and supportive supervisor messages. The primary and secondary outcomes were the Telework Environment Setup Behavior (12 items) and Somatic Symptom Scale-8 (SSS-8) scores, respectively. Analyses used a modified intention-to-treat approach with mixed-effects models.

**Results:**

Twelve clusters from 6 companies participated (182 and 188 in the intervention and control groups, respectively). No significant between-group differences were observed in environment setup (difference, 1.34; 95% CI, –1.67 to 4.35; *P* = .345) or SSS-8 (difference, –1.10; 95% CI, –2.59 to 0.38; *P* = .129) scores at follow-up. In the process evaluation, over half of intervention participants who completed the follow-up reported satisfaction, nearly 90% read the emails, and approximately 40% viewed the videos. Among video viewers, approximately two-thirds reported adopting recommended behaviors, while >80% rated the content useful.

**Conclusions:**

This secondary analysis did not detect statistically significant improvements in telework environment setup behaviors or somatic symptoms. Statistical conclusions regarding intervention effects remain inconclusive, underscoring the need for organizationally supported, multifaceted strategies to strengthen telework environments.

## Teaser

**Key points**
 

- **What is already known on this topic:** Since the COVID-19 pandemic, telework has rapidly expanded, raising concerns about musculoskeletal and other somatic symptoms associated with poor home-based work environments. Ergonomic interventions are effective in office settings; however, evidence regarding teleworkers is scarce. Previous studies have shown limited rigor, inadequate attention to contamination, a lack of theory-based design, and insufficient evaluation of somatic outcomes.
- **What this study adds:** This study was a secondary analysis of the TELEWORK cluster randomized controlled trial among employees of Japanese companies. The intervention included educational emails, short videos, feedback sheets, and supervisor messages based on behavioral science. No statistically significant improvements were observed in the environmental setup behaviors or somatic symptoms in the main analysis. However, process evaluation indicated that some participants engaged in the program and adopted the recommended behaviors. Overall, the statistical conclusions regarding the intervention’s effects remain inconclusive, although findings from the process evaluation suggest that some intervention components may be acceptable and potentially useful.
- **How this study might affect research, practice, or policy:** Education-centered interventions may require sufficient intervention exposure and organizational support to influence telework-related outcomes. Future studies should examine educational approaches combined with environmental supports, such as ergonomic equipment, and stronger organizational support to reinforce behavior change. In practice and policy, designing programs that enhance participant engagement and sustainability, supported structurally by employers and policymakers, may be important for protecting teleworkers’ health.

## 1. Introduction

Telework has rapidly expanded globally, particularly since the COVID-19 pandemic.^1^ Reported health consequences include musculoskeletal pain, gastrointestinal disturbances, fatigue, and sleep issues.^2^ The World Health Organization (WHO) and International Labour Organization (ILO) highlight telework-related ergonomic risks as a significant occupational health concern.^3^

Workspace quality is widely recognized as a central determinant of telework-related somatic symptoms. Ergonomic deficiencies—such as inappropriate chair and desk heights, poorly positioned monitors, and suboptimal lighting, temperature, and noise—are associated with musculoskeletal (eg, shoulder stiffness, low back pain) and nonmusculoskeletal problems (eg, headache, fatigue, impaired sleep, gastrointestinal discomfort, dizziness).^4–7^ Systematic reviews^8^^,^^9^ and a randomized controlled trial (RCT)^10^ involving office workers have indicated that ergonomic interventions, including adjustments to furniture and monitor placement and posture correction, can prevent or reduce these somatic symptoms. These findings highlight the practical importance of a sound workspace design for teleworkers as well.

Because teleworkers typically arrange their own remote workspaces, support for self-directed improvements is essential. While ergonomic interventions for office workers are well documented, studies explicitly addressing teleworkers' environment-setting behaviors remain limited.^11^ Nevertheless, evidence from conventional office settings suggests that brief ergonomics education combined with self-directed workstation adjustments can improve setup behaviors and reduce musculoskeletal symptoms, whereas effects on absenteeism and psychological outcomes appear limited.^12^ Limited telework evidence suggests that compact disc read-only memory–based ergonomic training improves knowledge, attitudes, and practices, but methodological limitations remain, including contamination across groups, lack of a theory-informed framework, and inadequate assessment of physical outcomes.^13^ Consequently, further evidence is required, preferably from cluster RCTs, to evaluate theory-driven interventions that encourage teleworkers to refine their work settings and to clarify the extent to which such behavioral changes prevent or alleviate somatic symptoms.

Against this backdrop, we conducted a cluster RCT (the TELEWORK study^14^) among teleworkers from multiple Japanese companies. This article reports a secondary analysis of this trial, focusing specifically on the outcomes related to telework environment setup behaviors and somatic symptoms. The primary outcome of the TELEWORK study was a change in step count and physical activity; telework environment setup behaviors and somatic symptoms were prespecified as secondary outcomes in the study protocol and trial registration (UMIN000053861). In addition, this secondary analysis incorporated key elements of the trial process evaluation to assess the participants’ engagement with and exposure to the intervention.

## 2. Methods

### 2.1 Study design and participants

This was a secondary analysis of the TELEWORK cluster RCT (UMIN000053861). The parent trial primarily reported changes in physical activity (step counts) as its primary outcome and found no statistically significant intervention effects.^15^ The present analysis focused on telework environment setup behaviors and somatic symptoms (Somatic Symptom Scale-8 [SSS-8]), which were prespecified as secondary outcomes in the trial registration and published protocol.^14^ The TELEWORK trial was designed as a multicomponent occupational lifestyle intervention targeting physical activity, musculoskeletal health, and improvements in the telework environment. In the current study, we specifically examined the components related to telework environment setup behaviors, with the aim of independently evaluating their effects.

This study used a cluster RCT design, with departments or corporate units as clusters assigned to either an intervention or control group. Clustering according to department was adopted to prevent contamination of the intervention effect, considering the potential for information sharing and behavioral influence within the same workplace. Each cluster comprised approximately 20-100 participants. All departments received identical interventions, and outcome evaluations were conducted at the individual level.

### 2.2 Eligibility and recruitment

Clusters were defined as workplace units where information diffusion was unlikely to cause cross-contamination. The target departments or offices were identified in coordination with representatives from the cooperating companies.

Eligible participants were expected to telework at least weekly. Exclusions included physician-restricted activity, anticipated long-term absence (eg, travel, retirement), pregnancy, or inability to return measuring instruments. Recruitment was facilitated by company representatives, and care was taken to avoid coercion or undue influence from supervisors. Employees within eligible clusters who met the inclusion criteria and provided informed consent were enrolled. Because telework frequency could change after recruitment, some participants reported teleworking less than once per week at baseline; however, these participants were retained in the main analysis according to the intention-to-treat principle.

### 2.3 Intervention

The intervention and evaluations were sequentially implemented by cluster from March 2024 to March 2025. As part of the broader TELEWORK study, the 12-week intervention was designed as a multicomponent occupational lifestyle program targeting physical activity, musculoskeletal health, and the telework environment through individual-, sociocultural-, physical-, and organizational-level strategies. This analysis focuses specifically on the components of this multilevel program aimed at promoting telework environment setup behaviors.

The 12-week program comprised individual- and department-level interventions. Coordinators forwarded 24 twice-weekly research team emails to participants, 5 of which addressed telework environment setup behaviors. Five short educational videos (2-4 minutes each) were used as on-demand learning tools. Drawing on previous studies,^4^ a technical brief,^3^ a checklist,^16^ and other resources (Appendix S1), the content addressed 12 behavioral actions related to 9 aspects of the physical home environment: lighting, display, keyboard, mouse, foot space, chair, temperature and humidity, ventilation, and noise. The videos incorporated behavioral science frameworks, including information-gap theory,^17^ dual-coding theory,^18^ and cognitive load theory^19^ to support behavioral change. Specifically, the videos featured (1) quizzes to introduce knowledge gaps, (2) visual aids alongside verbal explanations, and (3) no more than 3 recommended actions per video to minimize cognitive load. Additionally, personalized feedback sheets were provided based on the baseline telework environment setup behaviors and sensor data (2JCIE-BL01; Omron, Kyoto, Japan) measuring home temperature, humidity, illuminance, and noise levels compared with recommended ranges.

In 3 of the 24 emails, messages from department heads or senior executives encouraged program engagement, incorporating findings from feedback and contextual information about each department. The control group received no intervention during the study period, but was offered the same program after follow-up, following a wait-list design.

### 2.4 Outcome measures

Assessments were conducted via Google Forms. The baseline survey was administered approximately 3 weeks pre-intervention, and follow-up occurred immediately post-intervention. Because all data were self-reported, third-party assessors were uninvolved, and blinding was inapplicable. Furthermore, the intervention's nature precluded blinding participants to group allocation.

In this secondary analysis, telework environment setup behaviors were designated as the primary outcome and were assessed using an original 12-item scale developed based on previous studies,^4^ a technical brief,^3^ a checklist,^16^ and related resources (Appendix S1). The items assessed included the following: appropriate desk lighting, monitor height aligned with eye level, monitor distance of at least 40 cm (eg, length of an A3 sheet), resting forearms on the desk, using a user-friendly mouse, ensuring adequate foot space, using an ergonomically designed chair (adjustable seat height, backrest, armrests), adjusting chair height so feet rest naturally on the floor, maintaining comfortable room temperature and humidity, ensuring air ventilation, and avoiding disruptive noise. Responses were recorded on a 5-point Likert scale, ranging from 1 (strongly disagree) to 5 (strongly agree). Total scores ranged from 12 to 60, with higher scores indicating more desirable behaviors. Internal consistency, assessed using Cronbach α from baseline data (*n* = 310), yielded a coefficient of .83, supporting the conceptual coherence of the scale. The primary analysis metric was the adjusted between-group difference at post-intervention follow-up.

The SSS-8 score^20^ was used as a secondary outcome to evaluate potential improvements in somatic symptoms resulting from enhanced telework environment setup behaviors. The SSS-8, validated for use among Japanese adults, comprises 8 items that assess symptoms over the past week on a 5-point Likert scale: not at all (0), a little bit (1), somewhat (2), quite a bit (3), and very much (4). The total score (0-32) was treated as a continuous variable, and the analysis metric was the adjusted between-group difference at post-intervention follow-up.

### 2.5 Process evaluation

Process evaluation was conducted through the follow-up Google Forms survey, designed based on the “Reach” and “Implementation” components of the Reach, Effectiveness, Adoption, Implementation, and Maintenance framework.^21^ This process evaluation was administered only to participants in the intervention group. Items assessed included satisfaction with the comprehensive program (very satisfied/somewhat satisfied/neutral/somewhat dissatisfied/dissatisfied), email readership (read frequently [≥12 times]/read occasionally [<12 times]/did not read), feedback report review (reviewed the content/did not review the content), and several items specifically related to telework environment setup behaviors, including viewing of the telework-environment educational videos (viewed/did not view), behavioral actions taken after viewing (took some action/did not take any action), and perceived usefulness of the videos (very useful/somewhat useful/not very useful/not useful), all answered via multiple-choice format. To assess control group contamination, follow-up surveys asked these participants if they had become aware of the intervention emails' content (yes or no).

### 2.6 Additional variables

Additional variables assessed in the baseline survey via Google Forms included age, sex, household income, education, number of cohabitants, job type, working hours, telework frequency, psychological distress (K6^22^), body mass index (BMI), smoking, drinking frequency, and physical activity >1 hour per day.

### 2.7 Sample size calculation

The sample size was calculated based on the anticipated changes in the daily step count from the TELEWORK study,^14^ assuming Cohen *d* = 0.33, with a 5% type I error rate, 80% power, and an intraclass correlation coefficient of 0.02. To account for attrition, the required sample size was estimated to be 403, with a recruitment target of 500 participants. Because the sample size was powered for the parent trial's primary outcome (step count), this secondary analysis was not specifically powered for telework environment behaviors or somatic symptoms. As no prior studies quantified expected effect sizes, this analysis was exploratory; small effect sizes may have been undetectable.

### 2.8 Randomization and allocation procedures

The clusters were randomized in a 1:1 ratio to the intervention or control group. Allocation was stratified according to cluster size (<20 vs ≥20 participants) using stratified block randomization. A biostatistician at the University of Tsukuba generated the randomization list using a computerized random number table. The research team independently conducted the centralized registration and allocation. Allocation concealment was maintained until assignment, after which the allocation results were communicated via email to the investigators and company representatives.

Given the nature of the intervention, neither the participants nor intervention providers were blinded to the allocation. As the outcomes were self-reported by the participants, the outcome assessment was not blinded. In addition, data analysts were aware of the group allocation during statistical analyses.

### 2.9 Statistical analyses

The analyzed population included all participants who consented to participate in this study and completed the baseline survey (modified intention-to-treat [mITT]). Statistical analyses were performed using a mixed-effects model for repeated measures.

In the primary analysis, the fixed effects included group, time point, and group × time interactions with adjustments for covariates and stratification factors. The covariates consisted of baseline characteristics considered key prognostic factors for the outcomes—age (continuous), sex (male vs female), and baseline telework frequency (≥once/wk vs <once/wk)—and variables that showed baseline imbalances between groups: working hours (≥40 h/wk vs <40 h/wk) and BMI (≥25 vs <25). Additionally, cluster size (<20 vs ≥20 participants) was included as a factor.

To account for the cluster design, the workplace was used as the clustering variable, with random intercepts specified at the cluster level to account for intracluster correlation. The degrees of freedom and SEs were estimated using the Kenward-Roger method.

The primary outcome was the total score on the 12-item Telework Environment Setup Behavior Scale. The secondary outcome was the total SSS-8 score. Both models were analyzed using the same model specifications. To improve interpretability, the estimated marginal means and corresponding 95% CIs for each group × time point were also reported.

The following 6 analyses were conducted for both the primary and secondary outcomes: (1) the main analysis using the mITT population; (2) a sensitivity analysis excluding control participants who reported awareness of the intervention email content; (3) a subgroup analysis restricted to participants who teleworked at least once per week at both baseline and follow-up; (4) a per protocol analysis restricted to participants who teleworked at least once per week and, in the intervention group, read at least some of the intervention emails; (5) a per protocol analysis restricted to participants who teleworked at least once per week and, in the intervention group, reviewed the individualized feedback report; and (6) a per protocol analysis restricted to participants who teleworked at least once per week and, in the intervention group, viewed the telework-environment educational videos. For the control group in the subgroup and per protocol analyses, the same telework-frequency restriction was applied to ensure a comparable analytic population. No multiplicity adjustment was applied. A 2-sided significance level of 5% was used for all statistical tests. Process evaluation was conducted using data from the follow-up time point. All analyses were performed using IBM SPSS Statistics (version 29).

### 2.10 Ethical approval and informed consent

This study was approved by the Ethics Committee on Human Subjects of the Meiji Yasuda Health Foundation (approval no.: 2023-0002) and the Ethics Committee of the Faculty of Medicine of Teikyo University (approval no.: Teigai 23-042, external approval no.: 019). Informed consent was obtained after allocation, following a written explanation of the study’s objectives, content, and voluntary nature. This study adhered to the Declaration of Helsinki and relevant ethical guidelines for human research. A wait-list design ensured that participants in the control group received the intervention after the study completion, thereby allowing all participants to benefit from the intervention.

## 3. Results

### 3.1 Participant flow and analysis population

In total, 12 clusters from 6 companies were randomized, with 6 clusters (from 3 companies) assigned to the intervention group and 6 clusters (from 4 companies) to the control group. The intervention and control groups included 182 (mean cluster size = 30.3) and 188 participants (mean cluster size = 31.3), respectively, at the time of randomization.

After randomization, 60 participants were not included in the baseline analysis population: 39 did not provide consent or withdrew before baseline, 8 did not complete the baseline survey, and 13 company coordinators were excluded because they had prior knowledge of the intervention content. Consequently, the baseline analysis population comprised 310 participants, including 156 in the intervention group and 154 in the control group.

**Figure 1:**
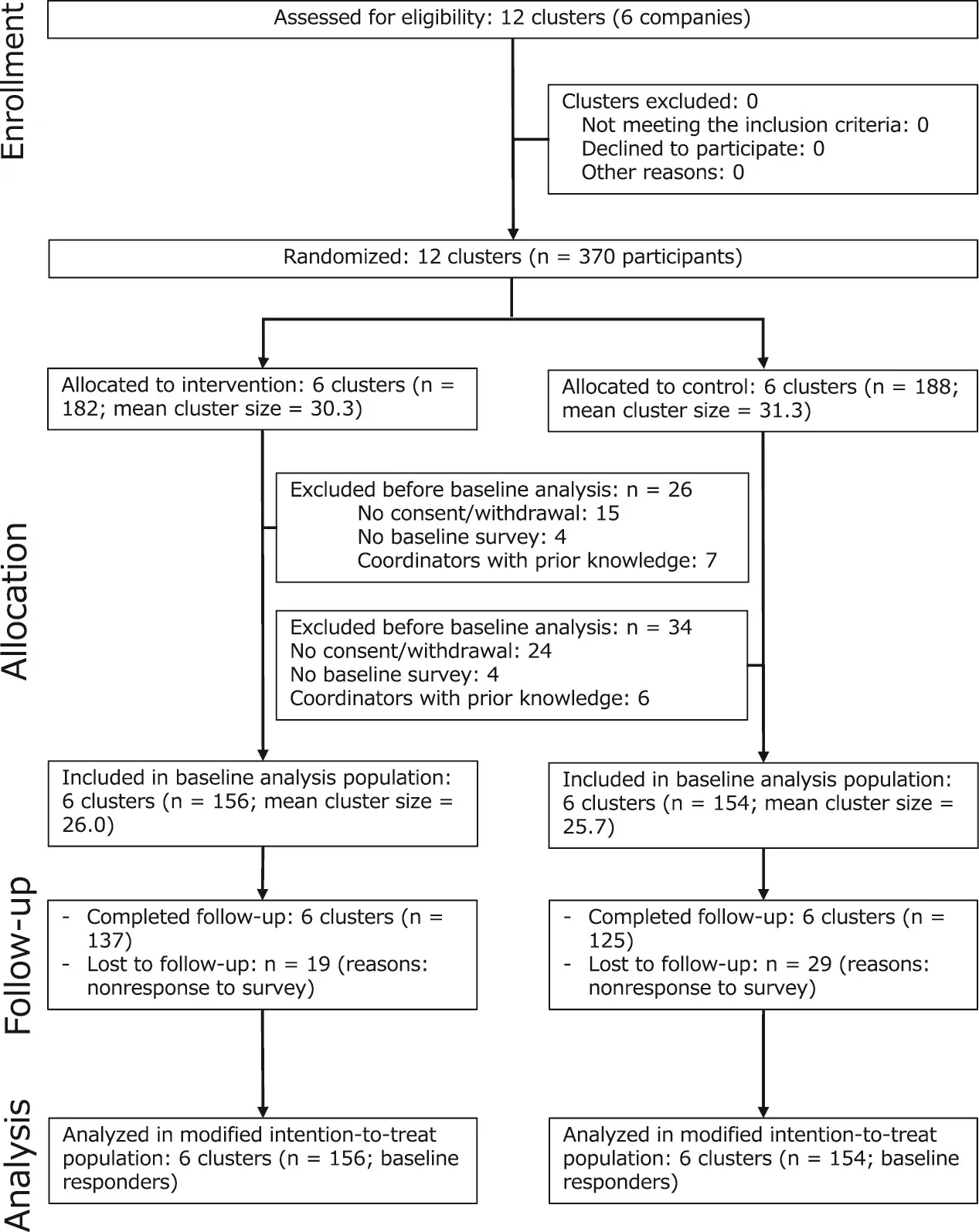
Participant flow diagram.

At follow-up, 137 intervention group participants and 125 control group participants completed the assessment; 19 and 29 participants, respectively, were lost to follow-up due to survey nonresponse. In accordance with the intention-to-treat principle, all participants who completed the baseline survey were included in the main analysis, and no additional participants were excluded from the analysis (Figure 1).

### 3.2 Baseline characteristics

Cluster- and individual-level baseline characteristics were generally comparable between groups (Table 1).

**Table 1:** Baseline characteristics of the participants according to group.

Variable	Intervention group	Control group
**Cluster-level characteristics**		
**Number of clusters**	6	6
**Number of companies**	3	4
**Mean (SD) cluster size, *n***	26.0 (8.5)	25.7 (9.1)
**Median cluster size (range), *n***	28.0 (13-35)	24.5 (16-40)
**Stratification factor (cluster size <20), *n***	1	2
**Cluster type (industry), *n***	Manufacturing: 2 Vehicle system development: 3 Information and communications: 1	Electrical equipment: 1 Printing and event-support services: 1 Vehicle system development: 3 Information and communications: 1
**Individual-level characteristics**		
**Number of participants**	156	154
**Age, mean (SD), y**	42.4 (12.0)	43.7 (11.4)
**Sex (male), *n* (%)**	120 (76.9)	105 (68.2)
**Household income ≥7 million JPY, *n* (%)**	93 (59.6)	88 (57.1)
**Education: college graduate or higher, *n* (%)**	123 (78.8)	124 (80.5)
**Number living with others (%)**	111 (71.2)	111 (72.1)
**Job type: professional occupations, *n* (%)**	114 (73.1)	116 (75.3)
**Work hours: ≥ 40 h/wk, *n* (%)**	131 (84.0)	116 (75.3)
**Telework frequency: ≥1 d/wk, *n* (%)**	111 (71.2)	116 (75.3)
**Psychological distress: K6 ≥5, *n* (%)**	73 (46.8)	73 (47.4)
**BMI ≥25, *n* (%)**	48 (30.8)	31 (20.1)

Abbreviations: BMI, body mass index; JPY, Japanese yen.

### 3.3 Primary outcome

The telework environment setup behavior scores at baseline were 46.57 (SD = 8.85) for the intervention group and 45.79 (SD = 8.35) for the control group. These scores increased at follow-up to 47.81 (SD = 8.11) and 46.49 (SD = 8.63), respectively (Table 2).

**Table 2:** Observed means of outcomes at baseline and follow-up according to group.

Outcome		Intervention		Control	
	Time point	*n*	Mean (SD)	*n*	Mean (SD)
**Telework environment setup behaviors**	Baseline	156	46.57 (8.85)	154	45.79 (8.35)
	Follow-up	137	47.81 (8.11)	125	46.49 (8.63)
	Change from baseline	137	1.23 (6.96)	125	0.86 (6.87)
**Somatic Symptom Scale-8 (SSS-8) score**	Baseline	156	6.86 (5.61)	154	7.86 (5.97)
	Follow-up	137	6.83 (5.19)	125	7.78 (5.29)
	Change from baseline	137	0.13 (4.47)	125	0.00 (5.33)

In the mITT analysis using a mixed-effects model, the estimated adjusted means at follow-up were 46.68 (95% CI, 44.03-49.32) in the intervention group and 45.34 (95% CI, 42.91-47.76) in the control group. The between-group difference was 1.34 (95% CI, –1.67 to 4.35; *P* = .345) (Table 3).

**Table 3:** Adjusted means and between-group differences (95% CIs) for primary and secondary outcomes.

Time point	Intervention	Control	Between-group difference (intervention − control)	*P* value ^a^	*P* value ^b^
**Telework environment setup behaviors**					
** Main analysis (modified intention-to-treat)**					
	*n* = 156	*n* = 154	–	–	–
** Baseline**	45.45 (42.81 to 48.09)	44.55 (42.16 to 46.94)	–	–	–
** Follow-up**	46.68 (44.03 to 49.32)	45.34 (42.91 to 47.76)	1.34 (−1.67 to 4.35)	.345	.602
** Sensitivity analysis (excluding contaminated controls)**					
	*n* = 156	*n* = 137	–	–	–
** Baseline**	45.43 (42.73 to 48.14)	44.42 (41.96 to 46.88)	–	–	–
** Follow-up**	46.66 (43.96 to 49.37)	45.23 (42.73 to 47.73)	1.43 (−1.67 to 4.53)	.330	.634
** Subgroup analysis (≥1 day telework)**					
	*n* = 88	*n* = 90	–	–	–
** Baseline**	44.93 (41.52 to 48.33)	44.27 (41.22 to 47.35)	–	–	–
** Follow-up**	46.94 (43.58 to 50.30)	44.80 (41.78 to 47.82)	2.14 (−1.78 to 6.06)	.249	.119
**Somatic Symptom Scale-8 (SSS-8) score**					
** Main analysis (modified intention-to-treat)**					
	*n* = 156	*n* = 154	–	–	–
** Baseline**	7.30 (5.89 to 8.72)	8.52 (7.23 to 9.81)	–	–	–
** Follow-up**	7.40 (6.00 to 8.81)	8.51 (7.20 to 9.81)	−1.10 (−2.59 to 0.38)	.129	.850
** Sensitivity analysis (excluding contaminated controls)**					
	*n* = 156	*n* = 137	–	–	–
** Baseline**	7.42 (5.92 to 8.93)	8.60 (7.22 to 9.98)	–	–	–
** Follow-up**	7.52 (6.03 to 9.01)	8.67 (7.28 to 10.07)	−1.15 (−2.78 to 0.48)	.148	.968
** Subgroup analysis (≥1 day telework)**					
	*n* = 88	*n* = 90	–	–	–
** Baseline**	6.40 (4.75 to 8.05)	8.23 (6.75 to 9.71)	–	–	–
** Follow-up**	6.80 (5.19 to 8.41)	8.47 (7.05 to 9.89)	−1.67 (−3.41 to 0.06)	.057	.822

^a^ *P* value for group comparison at follow-up period.

^b^ *P* value for interaction term between group and time.

As a sensitivity analysis excluding contaminated controls, the estimated adjusted means at follow-up were 46.66 (95% CI, 43.96-49.37) in the intervention group and 45.23 (95% CI, 42.73-47.73) in the control group. The between-group difference was 1.43 (95% CI, –1.67 to 4.53; *P* = .330).

In a subgroup analysis limited to participants teleworking at least 1 day per week, the between-group difference was 2.14 (95% CI, –1.78 to 6.06; *P* = .249).

In the per protocol analyses restricted to participants who teleworked at least once per week and were exposed to each intervention component, no statistically significant between-group differences at follow-up were observed for telework environment setup behavior scores (Table S1).

### 3.4 Secondary outcome

At baseline, the SSS-8 scores were 6.86 (SD = 5.61) and 7.86 (SD = 5.97) in the intervention and control groups, respectively. At the follow-up, the scores were 6.83 (SD = 5.19) and 7.78 (SD = 5.29), respectively (Table 2).

In the primary analysis, the between-group difference was –1.10 (95% CI, –2.59 to 0.38; *P* = .129) (Table 3). In the sensitivity analysis, the between-group difference was –1.15 (95% CI, –2.78 to 0.48; *P* = .148), whereas the subgroup analysis showed a similar pattern, with a difference of –1.67 (95% CI, –3.41 to 0.06; *P* = .057).

In the per protocol analyses, statistically significant between-group differences at follow-up were observed among feedback report reviewers and video viewers, with lower scores in the intervention group than in the control group (*P* = .012 and *P* = .007, respectively) (Table S1).

### 3.5 Process evaluation

Of the 137 participants in the intervention group, 72 (52.6%) were at least somewhat satisfied with the program. In total, 121 (88.3%) participants read periodic emails, 105 (76.6%) reviewed feedback reports, and 58 (42.3%) viewed educational videos. Of those who viewed the videos, 39 (67.2%) implemented the behaviors presented, and 50 (86.2%) rated the video as “useful” or “somewhat useful” (Table 4). In the control group, 125 participants responded to the contamination-check question at follow-up and 17 (13.6%) reported that they had become aware of the content of the emails distributed to the intervention group.

**Table 4:** Process evaluation among intervention participants at follow-up.

Process evaluation item	Respondents, *n*	Affirmative responses, *n*	%
**Program satisfaction (“satisfied” or “somewhat satisfied”)**	137	72	52.6
**Reading of regular emails (“read frequently” or “read occasionally”)**	137	121	88.3
**Checking feedback reports (“checked”)**	137	105	76.6
**Viewing of telework environment improvement videos (“viewed”)**	137	58	42.3
**Practiced behaviors introduced in the videos (“yes”)** ^**a**^	58	39	67.2
**Usefulness of the videos (“useful” or “somewhat useful”)** ^**a**^	58	50	86.2

^a^Items on practicing behaviors and perceived usefulness of the videos were asked only among participants who reported viewing the telework environment improvement videos.

## 4. Discussion

In this secondary analysis of a cluster RCT, we investigated the effects of a behavioral science–based educational intervention on telework environment setup behaviors and somatic symptoms in teleworkers. Across the mITT, sensitivity, subgroup, and per protocol analyses, statistically significant improvements in telework environment setup behaviors or somatic symptoms were not consistently detected, although some point estimates were in favorable directions. Because the sample size was calculated based on the primary outcome of the parent TELEWORK trial rather than the outcomes examined in the present analysis, the statistical results should be interpreted cautiously and should not be regarded as conclusive evidence of no intervention effect.

For telework environment setup behaviors, the estimated between-group difference was 1.34 points (95% CI, –1.67 to 4.35); while the point estimate leaned in a favorable direction for the intervention group, its quantitative clinical importance remains uncertain. Nonetheless, previous evidence suggests that poorer telework environment setup behaviors are associated with higher odds of somatic symptoms,^4^ supporting the relevance of this outcome to somatic symptom prevention. For SSS-8, the estimated between-group difference at follow-up was –1.10 points (95% CI, –2.59 to 0.38), also with the point estimate favoring the intervention group. The Japanese version of the SSS-8 has demonstrated good internal consistency and validity in a large general Japanese population.^20^ Although a recent systematic review suggested a minimal clinically important difference of 3 points for the SSS-8,^23^ this threshold provides only an approximate reference for interpreting between-group mean differences in a general teleworker population. Therefore, although the point estimates were in directions consistent with potential benefit, the practical or clinical significance of these estimates should be interpreted cautiously.

Previous studies have reported inconsistent outcomes of interventions that focus solely on information or educational support. For instance, computer-based ergonomics training for teleworkers demonstrated short-term improvements but had a high dropout rate of nearly 50%, and its long-term efficacy remains unclear.^13^ Research involving office workers has shown that physical interventions, such as workstation adjustments or provision of ergonomic chairs, can improve musculoskeletal symptoms,^9^^,^^10^ whereas the effects of educational interventions alone are inconsistent.^8^ In this study, a multifaceted intervention (on-demand videos, individualized feedback, senior management messages) yielded no statistically significant improvements in primary outcomes. While recent reviews associate telework with musculoskeletal symptoms, causal evidence from intervention studies remains scarce.^11^^,^^24^ Our findings provide novel data suggesting that education-centered interventions may require sufficient intervention exposure and organizational support to influence telework-related outcomes.

Several factors may explain the null findings. First, the on-demand educational videos may have lacked sufficient intensity. Given that the effects of educational ergonomics interventions on musculoskeletal symptoms have been inconsistent,^8^ the intervention used in this study may not have been sufficiently strong to induce behavioral or symptomatic changes. Second, the participants in the intervention group demonstrated relatively high baseline scores for telework environment setup behaviors, with a mean of 46.57, indicating that they had engaged in such behaviors to a considerable extent. Under such conditions, the additional impact of the intervention may have been minimal, and a ceiling effect could have hindered detection. These factors likely interacted to limit the observed effects.

The process evaluation further highlighted the issues associated with intervention adherence. Although many participants checked emails and feedback, video viewing rates were suboptimal and actual behavioral changes occurred only among a limited subset. This low adherence may have obscured the potential effects. Prior research has identified a lack of organizational support in telework settings as a health risk factor.^25^ In this context, messages from senior management may not have functioned as effective organizational support. However, some participants who viewed the videos reported perceived usefulness and behavioral changes, suggesting a potential benefit of educational content under certain conditions. Given the limited video viewing rate, the low level of intervention exposure may have attenuated the potential effects of the intervention. Therefore, the findings should not be interpreted as evidence that education-focused approaches are ineffective.

These findings have practical implications for supporting telework environments. Despite lacking significant between-group differences in the primary outcomes, the process evaluation suggests that improving participant engagement and intervention exposure is crucial for future programs. As emphasized in international guidelines from the WHO and ILO,^3^ organizational support from employers is important. To enhance adherence and foster behavioral change, comprehensive organizational strategies, including peer-driven social mechanisms, continuous support from occupational health professionals, and improvements to the physical work environment, may need to be combined with leadership messages.^26^

The key strengths of this study include its robust cluster RCT design, inclusion of multiple companies and a large employee sample, and development of an original scale to assess telework environment setup behaviors. The intervention was also practically applicable in real-world settings, enhancing its relevance for workplace implementation.

This study has limitations. Because these were secondary outcomes of the TELEWORK trial and sample size was powered for step count, statistical power to detect intervention effects here may have been limited. Therefore, caution is necessary when interpreting the results of statistical tests for intervention effect. Second, although the internal consistency of the newly developed scale was confirmed, further validation of its long-term validity and reproducibility is required. All outcomes were self-reported, and both the participants and analysts were aware of the group assignments, which could have introduced bias. Additionally, while the SSS-8 captures overall somatic burden, items like gastrointestinal symptoms or chest pain may not align with the intervention's expected mechanisms. Consequently, using the total SSS-8 score may have blunted the sensitivity to detect symptom changes directly linked to telework environment improvements. Third, the follow-up period was relatively short (12 weeks), precluding the assessment of long-term effects. Fourth, this study was limited to specific industries in Japan, and the generalizability to other sectors or countries should be approached with caution. The lack of statistically significant findings may also stem from failing to reach the target sample size and follow-up attrition. Finally, the post hoc per protocol analyses (based on feedback review and video viewing) may be subject to selection bias and should be interpreted as exploratory.

## 5. Conclusions

This exploratory, potentially underpowered secondary analysis found no statistically significant improvements in telework environment setup behaviors or somatic symptoms; thus, the findings regarding intervention efficacy remain inconclusive. The low level of intervention exposure, including the limited video viewing rate, may have attenuated the potential effects of the intervention, highlighting the need for higher-intensity, multifaceted organizational support and long-term evaluations in future studies.

## Supplementary Material

Supplementary_materials_uiag042

## Data Availability

The data used in this investigation were not accessible from a communal database because they encompassed information that may identify individuals or potentially sensitive participant data. In accordance with the ethical principles governing research in Japan, the Research Ethics Committee of Teikyo University imposed limitations on the distribution of the data collected during this study.
