# Supplementary material for: Effects of a telework environment intervention on work setup behaviors and somatic symptoms among Japanese teleworkers: a secondary analysis of the TELEWORK cluster randomized controlled trial
Source: J Occup Health. 2026 Jul 26;68(1):uiag042. doi: 10.1093/joccuh/uiag042 (PMC13502406; doi:10.1093/joccuh/uiag042)
Supplement: Supplementary file 1 [file supplementary_materials_uiag042.zip › Supplementary Appendix 1 20260724.docx]

Supplementary Appendix 1. References informing the intervention design

1. Bergefurt L, Appel-Meulenbroek R, Arentze T. How physical home workspace characteristics affect mental health: A systematic scoping review. *Work*. 2023;76(2):489-506. doi: 10.3233/WOR-220505
2. Buomprisco G, Ricci S, Perri R, De Sio S. Health and telework: new challenges after COVID-19 pandemic. *Eur J Environ Public Health*. 2021;5(2):em0073. doi:10.21601/ejeph/9705
3. Farbu EH, Skandfer M, Nielsen C, Brenn T, Stubhaug A, Höper AC. Working in a cold environment, feeling cold at work and chronic pain: a cross-sectional analysis of the Tromsø Study. *BMJ Open*. 2019;9:e031248. doi:10.1136/bmjopen-2019-031248
4. Hoe VCW, Urquhart DM, Kelsall HL, Zamri EN, Sim MR. Ergonomic interventions for preventing work-related musculoskeletal disorders of the upper limb and neck among office workers. *Cochrane Database Syst Rev*. 2018;(10):CD008570. doi:10.1002/14651858.CD008570.pub3
5. International Labour Organization. Teleworking arrangements during the COVID-19 crisis and beyond. Geneva: International Labour Office; 2021. Available from: https://www.ilo.org/publications/teleworking-arrangements-during-covid-19-crisis-and-beyond
6. Japan Human Factors and Ergonomics Society, (Ebara T and Yoshitake R (Eds.), Shimomura Y, Aoki K, Kotani K, Toriizuka T, et al.). Seven practical human factors and ergonomic tips for teleworking/home-learning using tablet/smartphone devices, first edition, Japan Human Factors and Ergonomics Society, 2020.
7. Japan Society for Office Studies. *Zaitaku waaku ni okeru ningen kougakuteki gaidorain ~Ver.1.0~*. Japan Society for Office Studies; 2021. Available from: https://www.jos-japan.jp/activity/pdf/humanengineering_2021.pdf
8. Jun D, Michaleff Z, Johnston V, O’Leary S. Physical risk factors for developing non-specific neck pain in office workers: a systematic review and meta-analysis. *Int Arch Occup Environ Health*. 2017;90:373–410. doi:10.1007/s00420-017-1205-3
9. Kanamori S, Tabuchi T, Kai Y. Association between the telecommuting environment and somatic symptoms among teleworkers in Japan. *J Occup Health*. 2024;66(1):uiad014. doi:10.1093/joccuh/uiad014
10. Korhonen T, Ketola R, Toivonen R, Luukkonen R, Häkkänen M, Viikari-Juntura E. Work related and individual predictors for incident neck pain among office employees working with video display units. *Occup Environ Med*. 2003;60:475–482. doi: 10.1136/oem.60.7.475
11. Larrea-Araujo C, Ayala-Granja J, Vinueza-Cabezas A, Acosta-Vargas P. Ergonomic risk factors of teleworking in Ecuador during the COVID-19 pandemic: a cross-sectional study. *Int J Environ Res Public Health*. 2021;18(10):5063. doi:10.3390/ijerph18105063
12. Lee S, de Barros FC, de Castro CSM, de Oliveira Sato T. Effect of an ergonomic intervention involving workstation adjustments on musculoskeletal pain in office workers — a randomized controlled clinical trial. *Ind Health*. 2021;59(2):78–85. doi:10.2486/indhealth.2020-0188
13. Marcus M, Gerr F, Monteilh C, Ortiz DJ, Gentry E, Cohen S, et al. A prospective study of computer users: II. Postural risk factors for musculoskeletal symptoms and disorders. *Am J Ind Med*. 2002;41(4):236–249. doi:10.1002/ajim.10067
14. Matsugaki R, Muramatsu K, Tateishi S, Nagata T, Tsuji M, Hino A, et al. Association between telecommuting environment and low back pain among Japanese telecommuting workers: a cross-sectional study. *J Occup Environ Med*. 2021;63(12):e944–e948. doi:10.1097/JOM.0000000000002412
15. Ministry of Health, Labour and Welfare. *Terewaaku wo okonau roudousha no anzeneisei wo kakuhosurutame no chekkurisuto (jigyousha-you)*. 2021. Available from: https://www.mhlw.go.jp/content/000755113.pdf
16. Ministry of Health, Labour and Welfare. *Shokuba ni okeru youtsuu yobou taisaku shishin*. 2013. Available from: https://www.mhlw.go.jp/content/11303000/001376471.pdf
17. Van Niekerk SM, Louw QA, Hillier S. The effectiveness of a chair intervention in the workplace to reduce musculoskeletal symptoms: A systematic review. *BMC Musculoskelet Disord*. 2012;13:145. doi:10.1186/1471-2474-13-145
18. Saito S, Piccoli B, Smith MJ, Sotoyama M, Sweitzer G, Villanueva MBG, Yoshitake R. Ergonomic guidelines for using notebook personal computers. *Ind Health*. 2000;38(4):421–434. doi: 10.2486/indhealth.38.421
19. Sato H, Matsugaki R, Ando H, Odagami K, Mafune K, Tateishi S, et al. A cross-sectional study of the association between telecommuting environments and shoulder pain among Japanese telecommuting workers. *J Occup Environ Med*. 2023;65(3):e101–e104. doi:10.1097/JOM.0000000000002765
20. Xiao Y, Becerik-Gerber B, Lucas G, Roll SC. Impacts of working from home during COVID-19 pandemic on physical and mental well-being of office workstation users. *J Occup Environ Med*. 2021;63(3):181–190. doi:10.1097/JOM.0000000000002097
